# Contemporary experience of polyhydramnios: A single‐centre experience

**DOI:** 10.1002/ajum.12247

**Published:** 2021-05-26

**Authors:** Christopher Kyriacou, Louise Roper, Stephanie Mappouridou, Christoph Lees, Tomas Prior

**Affiliations:** ^1^ Tommy's National Centre for Miscarriage Research Queen Charlotte's & Chelsea Hospital Imperial College London UK; ^2^ Institute for Reproductive and Developmental Biology Imperial College London UK; ^3^ Centre for Fetal Care Queen Charlotte's & Chelsea Hospital Imperial College London UK

**Keywords:** polyhydramnios, fetal abnormality, pregnancy, fetal monitoring, ultrasound

## Abstract

**Introduction:**

Polyhydramnios is common; the majority of cases are idiopathic, but maybe associated with fetal abnormality. Literature suggests the volume of amniotic fluid discriminates idiopathic from pathological polyhydramnios but is not unanimous. We assessed fetal anomaly incidence amongst women with polyhydramnios and the role of discriminatory variables in identifying pathological cases.

**Methods:**

Retrospective observational cohort study at an inner‐city London fetal medicine centre. Records for patients referred and/or diagnosed with polyhydramnios were reviewed as well as maternal/fetal demographics, amongst singleton pregnancies using the Astraia™ database from January 2015–2016. Estimated fetal weight was calculated using the Hadlock model (biometry undertaken at diagnosis). Student's t‐test/one‐way ANOVA compared means; chi‐squared tests compared proportions.

**Results:**

120 cases were identified. 36 (30%) had fetal abnormality. There was no difference in AFI between fetuses with an abnormality and without (26.7 vs 25.2 cm, P = 0.22). AFI was normalised for weight (AFI (cm)/estimated fetal weight (kg)): AFI/kg was significantly different between cases with fetal abnormality and without (24.4 vs 16.7 cm/kg, P < 0.001) – incidence of abnormality increased with increasing AFI/kg (P = 0.007). Early gestational diagnosis was associated with higher rates of anomaly (P = 0.004). Differences in AFI/kg between those with and without abnormality were not significant when adjusted for gestation. AFI was significantly higher in cases of abnormality diagnosed at later gestation (P = 0.005).

**Conclusion:**

Excess volume of amniotic fluid alone does not denote abnormality. Earlier gestations and higher AFI/kg corresponded with significantly increased rates of anomaly. However, the latter is a result of confounding by gestation, which is closely correlated with fetal weight.

## Introduction

Polyhydramnios is a relatively frequent abnormality of pregnancy with an incidence of 0.9% to 3.9% in reported studies.^1^, ^2^ Polyhydramnios may be defined as an amniotic fluid index (AFI) of greater than 25 cm, a deepest vertical pool (DVP) of greater than 8 cm or by gestation specific Moore and Cayle nomograms.^3^, ^4^ The identification of polyhydramnios can be distressing for parents and challenging for clinicians as, while the majority of cases are idiopathic, polyhydramnios may be associated with significant fetal anomaly or maternal disease. Therefore, appropriate counselling of patients without causing undue distress can be difficult.

The degree of polyhydramnios is often reported as mild (25–30 cm), moderate (30.1–35 cm) or severe (>35.1 cm), and studies have suggested a correlation between the degree of polyhydramnios and the likelihood of fetal anomaly.^5^ However, clinically, it remains unclear as to whether cases of polyhydramnios should be stratified based on amniotic fluid index or not. Appropriate stratification would be valuable in identifying cases requiring further investigation and for counselling discussions with patients.

From the second trimester onward, amniotic fluid is predominantly a product of the fetal kidneys, and a pathological accumulation of amniotic fluid may result from increased fetal urine production. While urine output is accepted to vary according to weight in both adults and children, consideration of fetal weight when evaluating cases of polyhydramnios is not routine practice. While the relationship between fetal weight and amniotic fluid volume has been studied by some authors and suggested to be of little value,^6^, ^7^, ^8^ the populations investigated comprised low‐risk cases, amongst whom there is considerable variation in ‘normal’ amniotic fluid volumes. To our knowledge, the value of adjustment for fetal weight in a population with abnormal amniotic fluid volume is yet to be considered.

In this retrospective observational cohort study, we reviewed cases of polyhydramnios referred to a tertiary referral fetal medicine unit over a one‐year period and reported the incidence of associated fetal anomaly in relation to AFI, AFI normalised for estimated fetal weight (EFW), and AFI at specific pregnancy gestation time points.

## Methods

This retrospective observational cohort study was performed at a single inner‐city specialist maternity hospital in London with a tertiary referral fetal medicine service. All cases referred to our centre (either from our own scan department or from other hospitals) as polyhydramnios following routine ultrasound or clinical assessment (with or without additional fetal anomaly) were seen by a fetal medicine specialist in the centre for fetal care (CFC) for further evaluation.

Relevant case records were identified via a search of the CFC Astraia (Astraia GMBH‐Munich)™ database over a one‐year period from January 2015. Search terms included a ‘diagnosis of’, or ‘referral for’, ‘polyhydramnios’, an AFI documented as >25 cm or >95th centile or a DVP documented as >8 cm. All cases of multiple pregnancy were excluded. Pregnancies included were all dated via a 1st trimester crown‐rump length (CRL). Electronic case records were reviewed, and maternal demographics, estimated fetal weight, presence of maternal diabetes, presence of structural, chromosomal/genetic or Doppler abnormalities were recorded. Estimated weight centiles were calculated and documented for each case.

Each case was then reviewed and classified accordingly by a fetal medicine specialist as polyhydramnios with no fetal anomaly, or polyhydramnios with fetal anomaly. Those found to have AFI <25 cm or DVP <8 cm on assessment in the CFC were not excluded as they had met the criteria on assessment prior to referral. In these cases, AFI was then found to be at the upper limit of normal. AFI is known to fluctuate throughout pregnancy. Pathological cases were then further sub‐classified according to abnormality identified. All babies delivered at our centre were reviewed postnatally to confirm their antenatal assessment.

### Statistics

Student’s t‐test and one‐way ANOVA were used to compare mean values of continuous variables between groups, while chi‐squared test was used to compare proportions. Significance was classed as having a P value of 0.05 or less.

### Ethical approval

This study involved retrospective review of case notes. As such, it was performed as a local clinical audit as it was designed to produce information to inform on delivery of best care.

## Results

Over a one‐year period, 120 cases with polyhydramnios were identified from the database. After specialist fetal medicine review, 36 (30%) were diagnosed with a fetal abnormality.

Maternal demographics and scan details at initial fetal medicine assessment are documented in Table 1. The majority of cases assessed had an AFI of 20–35 cm (111 cases – 92.5%). The incidence of maternal diabetes was 15.8%. There were significantly more cases of diabetes in the group without fetal abnormality (21.4% vs 5.6% with fetal abnormality, P = 0.03).

**Table 1:** Maternal demographics and scan details at initial fetal medicine assessment (mean and range)

Age	32.8 (18–46)
BMI (kg/m^2^)	28.2 (16–45)
Gestation at diagnosis (weeks)	28.7 weeks (28–39)
Estimated weight at diagnosis (g)	1999.7 g (403–4290)
Estimated weight per centile at diagnosis	48.3 (2–98)
AFI at diagnosis (cm)	26.2 (16–48)

Cases were categorised according to presence or absence of a fetal abnormality. AFI was compared between fetuses with a fetal abnormality (mean AFI = 26.7 cm) and those without (mean AFI = 25.2 cm). No significant difference in mean AFI was observed (P = 0.22). This absence of significant variation remained when AFI values were expressed as multiples of the median (MoM) for each gestational age group (1.77 MoM vs. 1.87 MoM, P = 0.12). Twenty cases had an AFI greater than 30 cm at diagnosis of polyhydramnios (16.7%). The incidence of fetuses with an AFI of >30 cm was not significantly different between those with and without a fetal abnormality (17.7% vs 15.0%, P = 0.79).

**Figure 1:**
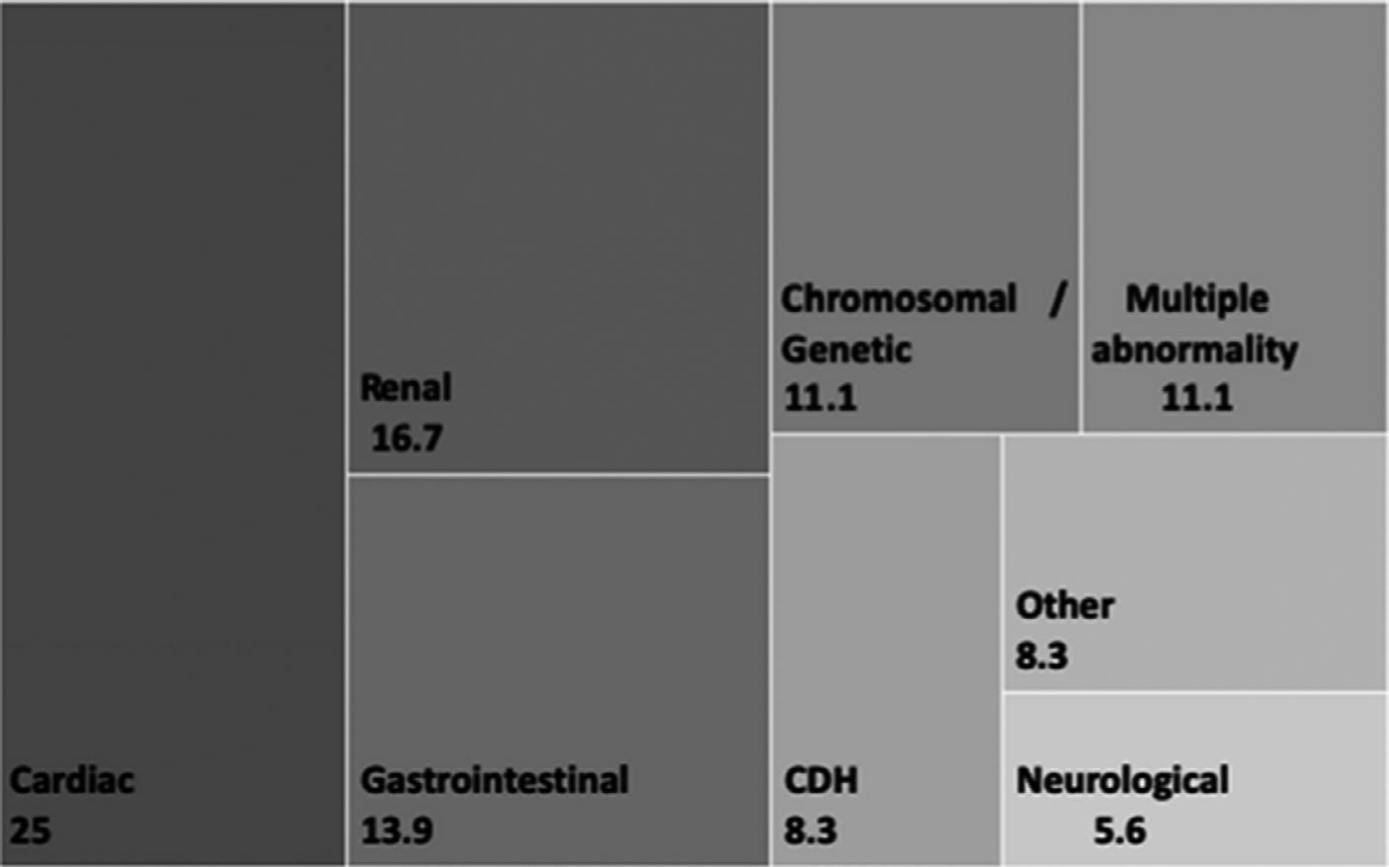
Antenatally detected fetal abnormalities in polyhydramnios (%)

The sub‐type of abnormality is reported in Figure 1. A number of different sub‐types of fetal abnormality were identified with the most common being cardiac (10 cases, 27.8%) and renal (6 cases, 16.7%). No significant variation in the mean AFI across the subgroups was observed.

**Figure 2:**
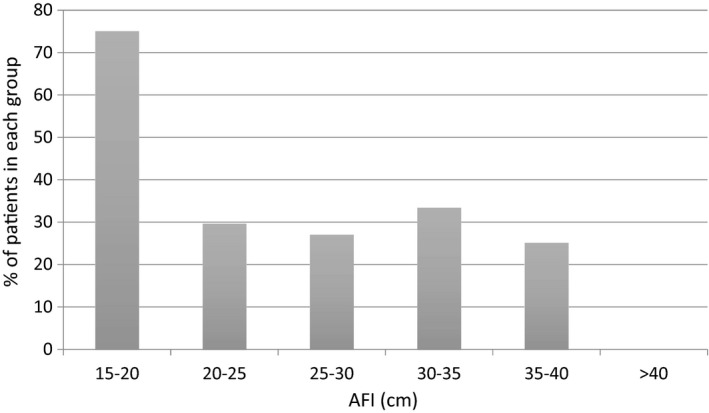
Fetal abnormality (%) according to AFI (cm)

Cases were categorised according to AFI from 15–20 cm, in 5 cm increments, to AFI greater than 40cm. The data are presented in Figure 2. Although a high proportion of fetal abnormality is seen with an AFI of 15–20 cm, this group does not have polyhydramnios and included 3 out of 4 total cases. The remaining 116 cases had an AFI above 20 cm. Those with AFI < 25 cm were included as a baseline comparison for interest. When the incidence of fetal abnormality was compared between the AFI groupings, no significant variation was observed (P = 0.66).

Other ultrasound characteristics were compared between cases with and without fetal anomaly. Differences were observed in estimated fetal weight and gestation at diagnosis, but not estimated fetal weight centile (Table 2).

**Table 2:** Ultrasound characteristics in normal and abnormal fetuses (mean with P value)

	Normal	Abnormal	P value
AFI (cm)	25.36	26.6	0.18
Estimated fetal weight (g)	2194	1549	<0.001
Estimated fetal weight per centile	49	46	0.29
Gestation at diagnosis (weeks)	32.27	28.95	<0.001

**Figure 3:**
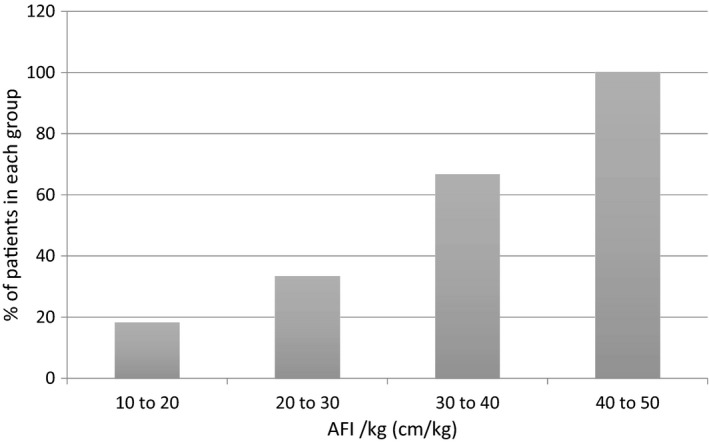
Incidence of fetal abnormality (%) in cases diagnosed with polyhydramnios according to AFI/kg (cm/kg)

To further examine the impact of fetal weight, AFI was normalised for weight (AFI (cm)/EFW (kg)). The AFI/kg differed between cases with a fetal abnormality and those without (24.4 vs. 16.7, P = <0.001). Cases were categorised according to AFI/Kg, and the incidence of fetal abnormality compared in Figure 3. Significant variation was observed, with increasing incidence of fetal abnormality as the AFI/Kg increased (P = 0.007). 18% of those with an AFI/kg of 10–20 cm/kg had a confirmed fetal abnormality, far lower than the incidence of fetal abnormality in cases with AFI/kg of 40 to 50 cm/kg, which was 100%.

**Figure 4:**
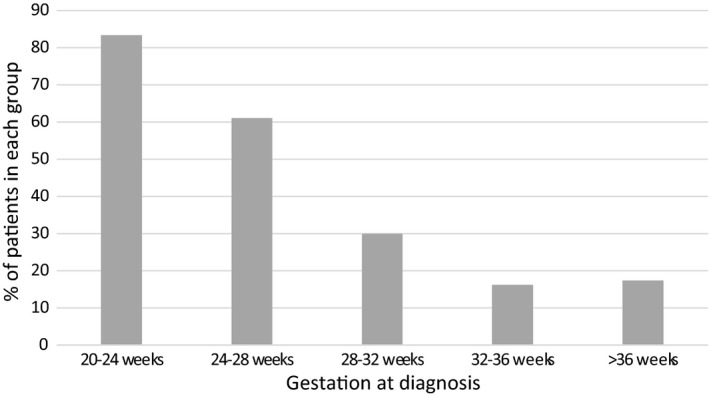
Incidence of fetal abnormality (%) according to gestation at diagnosis of polyhydramnios

The impact of gestation was then assessed, and the results are presented in Figure 4. The incidence of fetal abnormality in cases diagnosed with polyhydramnios at differing gestations was compared. Diagnosis at early gestations was associated with a significantly increased rate of fetal anomaly (P = 0.004). A diagnosis of polyhydramnios at 20–24 weeks was associated with fetal abnormality in 83% of cases, compared with just 16–17% in the group diagnosed at either 32–36 weeks or after 36 weeks.

**Figure 5:**
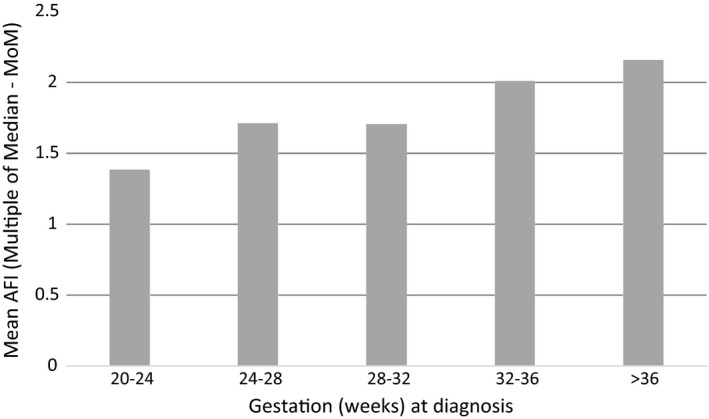
Mean AFI (MoM) in fetuses with abnormality, according to gestation at diagnosis

To determine the significance of gestation and estimated fetal weight as inter‐dependent variables on the presence/absence of fetal abnormality cases were sub‐classified according to gestation at diagnosis (20–24, 24–28, 28–32, 32–36 and >36 weeks). AFI was found to be significantly higher when cases of fetal abnormality were diagnosed at a later gestation (P = 0.005). We examined mean AFI multiples of the median (MoM) within each gestation group to compare the groups further, highlighted in Figure 5. Mean AFI MoM in cases diagnosed with a fetal abnormality at later gestations had a greater deviation from the median (P = 0.001). However, no significant difference was observed in AFI when comparing normal and abnormal cases in each gestation sub‐group.

Finally, we calculated 50th centile values for AFI/Kg for each gestation point using Moore and Cayle AFI nomograms and Hadlock estimated fetal weight nomograms. The AFI/Kg of each case at the time of diagnosis was then compared to the 50th centile AFI/kg for that gestation and expressed as MoM. By doing this, we found no significant difference in the AFI/kg between cases with an abnormality and those without (1.97MoM vs. 2.12MoM, P = 0.097).

**Figure 6:**
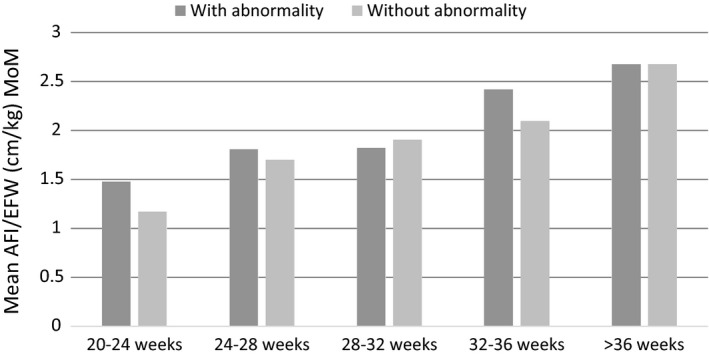
Mean AFI/kg MoM in fetuses with abnormality according to gestation at diagnosis

We then subdivided AFI/kg MoM into gestation groups and plotted the means of each on Figure 6. Mean AFI/kg MoM in those with a fetal abnormality is significantly higher at a later gestation (>36 weeks) compared to those at 20–24 weeks (P = 0.001), 24–28 weeks (P = 0.0154) and 28–32 weeks (P = 0.0282). However, the mean AFI/kg MoM difference in each gestation sub‐group between those with a fetal abnormality and those without is not significant (P = 0.1854).

## Discussion

A diagnosis of polyhydramnios can result in uncertainty for both patient and doctor, with a wide range of potential causes and marked variability in potential impact on the pregnancy. The rate of fetal abnormality observed in this study cohort was 30%. The reported incidence of fetal abnormality in published studies varies from 11% in one large retrospective study^9^ to as much as 91% in cases of moderate to severe polyhydramnios.^10^

Intuitively, increasing deviation of amniotic fluid volume from normal limits might be expected to result in increasing rates of fetal abnormality, a suggestion that is supported by published literature.^10^, ^11^ However, findings from this study suggest that the degree of amniotic fluid excess alone does not correlate with the presence of fetal abnormality. It is possible that a degree of selection bias is responsible, for example some cases of mild polyhydramnios with no other evidence of fetal abnormality not being referred for fetal medicine assessment, despite this being the local policy. However, within the study cohort, there were 44 cases with an AFI 20–25 cm. The incidence of fetal abnormality in this group was 29% (31/44), higher than that reported in other case series and consistent with the incidence in the overall study cohort. This finding is important as it suggests that all cases of polyhydramnios, even those where amniotic fluid volumes are only mildly elevated, require consideration of the presence of fetal abnormality.

A wide variety of fetal abnormalities were observed including cardiac, renal, gastrointestinal, chromosomal/genetic. Amniotic fluid volumes did not show significant variation between the sub‐groups. This reiterates the importance of thorough examination of all fetal anatomy, no matter the severity of polyhydramnios.

When cases with and without antenatally detected fetal abnormality were compared, gestation at referral and EFW varied significantly between the two groups.

Comparing the incidence of fetal abnormality at each gestation demonstrated that diagnosis at earlier gestations was associated with a significantly increased incidence of fetal anomaly, with a step wise reduction in incidence with advancing gestation. High rates of abnormality have been previously reported in cases of polyhydramnios diagnosed at early gestations.^12^ This highlights the importance of thoroughly investigating polyhydramnios identified at the anomaly ultrasound scan, whether it be mild, moderate or severe.

Polyhydramnios at earlier gestations was associated with lower AFI values and a smaller deviation from normality when compared to cases diagnosed at later gestations. A larger deviation from normality in those diagnosed with polyhydramnios at later gestations is particularly important for counselling women diagnosed after 32 weeks where, occurring on the background of normal antenatal care, it can cause significant distress. In this data set, cases of polyhydramnios diagnosed after 32 weeks’ gestation with an AFI MoM of <2 had an incidence of fetal abnormality of 9.9% (4/45), compared to 33.3% (7/21) in those with an AFI MoM > 2. Cases diagnosed at <32 weeks’ gestation with an AFI MoM < 2 had an abnormality rate of 51.1% (23/45). This indicates that with polyhydramnios identified closer to term, abnormalities would only be suspected if polyhydramnios was severe.

When AFI was normalised for fetal weight, higher AFI/kg was associated with significantly increased rates of fetal anomaly. However, this association appears as a result of confounding by gestation, which is of course closely correlated to fetal weight. When cases were subdivided according to gestation, no difference in AFI/kg MoM was observed between cases with and without a fetal abnormality within each gestation group. These data support previous assertions that adjustment for fetal weight is not required when evaluating cases of polyhydramnios.^6^, ^7^, ^8^

This study reports a series of polyhydramnios cases from a single inner‐city specialist maternity hospital in London with a tertiary referral fetal medicine unit over one year. Allowing for limitations due to its retrospective nature, data from studies such as this can facilitate appropriate counselling of patients following a diagnosis of polyhydramnios. While AFI alone does not appear to be a good discriminator of the presence/absence of fetal abnormality, gestation at diagnosis does have a significant relationship. At early gestations, even mild polyhydramnios is strongly associated with fetal abnormality whereas in contrast, at later gestations, fetal abnormality appears to be associated predominantly with greater deviations from normal amniotic fluid volumes.

These data suggest that all cases of polyhydramnios require careful assessment to rule out associated fetal abnormality, and facilitate appropriate counselling, particularly in late 3rd trimester cases, where associated abnormality is unlikely without severe polyhydramnios.

## Authorship statement

The authorship conforms with the journal’s authorship policy, and all authors are in agreement with the content of the submitted manuscript.

## Funding

CK, LR, SM and TP are supported by Imperial College Healthcare NHS Trust. CL is supported by the NIHR Biomedical Research Center based at Imperial College Healthcare NHS Trust and Imperial College London. The views expressed are those of the author(s) and not necessarily those of the NHS, the NIHR or the Department of Health.

## Conflict of interest statement

All authors have no conflicts of interest to declare.

## Author Contributions

**Christopher Kyriacou:** Conceptualisation (equal); Data curation (equal); Formal analysis (equal); Investigation (equal); Methodology (equal); Project administration (equal); Writing‐original draft (equal); Writing‐review & editing (equal). **Louise Roper:** Data curation (equal); Investigation (equal); Methodology (equal); Project administration (equal); Writing‐original draft (equal); Writing‐review & editing (equal). **Stephanie Mappouridou:** Data curation (equal); Investigation (equal); Methodology (equal); Writing‐original draft (equal); Writing‐review & editing (equal). **Christoph Lees:** Conceptualisation (equal); Formal analysis (equal); Methodology (equal); Resources (equal); Supervision (equal); Writing‐original draft (equal); Writing‐review & editing (equal). **Tomas Prior:** Conceptualisation (lead); Data curation (equal); Formal analysis (lead); Investigation (equal); Methodology (equal); Project administration (equal); Writing‐original draft (equal); Writing‐review & editing (equal).
